# Chemical and cellular oxidant production induced by naphthalene secondary organic aerosol (SOA): effect of redox-active metals and photochemical aging

**DOI:** 10.1038/s41598-017-15071-8

**Published:** 2017-11-09

**Authors:** Wing Y. Tuet, Yunle Chen, Shierly Fok, Dong Gao, Rodney J. Weber, Julie A. Champion, Nga L. Ng

**Affiliations:** 1 School of Chemical and Biomolecular Engineering, Georgia Institute of Technology, Atlanta, GA United States; 2 School of Materials Science and Engineering, Georgia Institute of Technology, Atlanta, GA United States; 3 School of Civil and Environmental Engineering, Georgia Institute of Technology, Atlanta, GA United States; 4 School of Earth and Atmospheric Sciences, Georgia Institute of Technology, Atlanta, GA United States

## Abstract

Exposure to air pollution is a leading global health risk. Secondary organic aerosol (SOA) constitute a large portion of ambient particulate matter (PM). In this study, the water-soluble oxidative potential (OP) determined by dithiothreitol (DTT) consumption and intracellular reactive oxygen and nitrogen species (ROS/RNS) production was measured for SOA generated from the photooxidation of naphthalene in the presence of iron sulfate and ammonium sulfate seed particles. The measured intrinsic OP varied for aerosol formed using different initial naphthalene concentrations, however, no trends were observed between OP and bulk aerosol composition or seed type. For all experiments, aerosol generated in the presence of iron-containing seed induced higher ROS/RNS production compared to that formed in the presence of inorganic seed. This effect was primarily attributed to differences in aerosol carbon oxidation state ${\boldsymbol{(}}{\bar{{\bf{O}}{\bf{S}}}}_{{\bf{c}}}{\boldsymbol{)}}$. In the presence of iron, radical concentrations are elevated via iron redox cycling, resulting in more oxidized species. An exponential trend was also observed between ROS/RNS and ${\boldsymbol{(}}{\bar{{\bf{O}}{\bf{S}}}}_{{\bf{c}}}{\boldsymbol{)}}$ for all naphthalene SOA, regardless of seed type or aerosol formation condition. This may have important implications as aerosol have an atmospheric lifetime of a week, over which ${\boldsymbol{(}}{\bar{{\bf{O}}{\bf{S}}}}_{{\bf{c}}}{\boldsymbol{)}}$ increases due to continued photochemical aging, potentially resulting in more toxic aerosol.

## Introduction

Air pollution exposure ranks among the top ten global human health risks^1^ with multiple epidemiological studies reporting associations between various cardiopulmonary health effects, elevated particulate matter (PM) concentrations^1–8^, and particle oxidative potential (OP)^9–12^. Toxicological studies suggest PM-induced oxidant production as a possible mechanism linking PM exposure and observed health effects^13–16^. Multiple chemical and cellular assays have been developed and utilized to measure PM-induced oxidant production. For instance, cell-free chemical assays that utilize an antioxidant to simulate biologically relevant redox reactions and ultimately measure the redox potential of PM^17,18^ and cellular assays that employ a probe capable of reacting with reactive oxygen and nitrogen species (ROS/RNS) produced as a result of PM exposure^19,20^ have been developed. Both types of assay have been used in prior studies to elucidate chemical species associated with oxidant production^9,20–31^. Despite these efforts, the specific constituents responsible for the overall health effects induced by PM exposure remain unclear as ambient mixtures are complex.

Organic aerosol constitute a significant portion of ambient PM^32,33^, and multiple field studies have repeatedly shown that secondary organic aerosol (SOA, formed from the oxidation of volatile organic compounds in the atmosphere) often dominate over aerosol of primary origin (e.g., aerosol emitted directly from combustion engines), even in urban centers^33–35^. While there have been several recent studies regarding the health effects of SOA^36–45^, there are still important gaps in knowledge that have not been addressed. For instance, organic aerosol have a lifetime of approximately one week^46^; continued photochemical aging can alter the chemical and physical properties of aerosol, which may have implications on resulting health effects. These potential effects have not been fully explored as the majority of current studies have focused on freshly formed SOA^36,45,47–49^. In addition, the presence of redox-active metals on SOA health effects have not been considered even though laboratory studies have shown that the presence of metal-containing seeds influences SOA formation and chemical composition^50–53^, and these metals are readily emitted via various processes (e.g., traffic, mechanical processes, combustion)^22,54^. Furthermore, redox-active metals such as iron may participate in redox cycling, as well as Fenton-like reactions^55,56^. These reactions produce radicals capable of enhancing the degree of oxidation of organic aerosol when internally mixed with organic aerosol, resulting in stronger oxidizing agents that may induce more ROS/RNS production upon cellular exposure^49^. Depending on the source, iron may exist in either coarse or fine mode, with a majority in the coarse mode and a small fraction in the fine mode^57–59^. As such, there exists some overlap between the size distributions of iron and submicron organic aerosol, which is sufficient for iron to serve as a catalyst in Fenton-like reactions in some fraction of the organic aerosol.

In the present study, naphthalene photooxidation SOA were generated in the presence of metal-containing (iron (II) sulfate, FS) and inorganic (ammonium sulfate, AS) seed. For both seed types, a series of laboratory chamber experiments with different initial naphthalene concentrations was conducted to produce aerosol of various degrees of oxidation. Multiple samples were also collected from a single experiment to obtain aerosol of different photochemical age. Oxidant production was measured using chemical and cellular assays (i.e., water-soluble OP as determined by dithiothreitol (DTT) consumption^21^ and intracellular ROS/RNS production as detected using carboxy-H_2_DCFDA^20^). Tuet *et al*.^45,49^ recently investigated the water-soluble oxidative potential and cellular ROS/RNS production for SOA formed from common biogenic and anthropogenic precursors. Here, we choose to focus on naphthalene SOA as it was shown to have the highest response among different SOA systems previously studied in Tuet *et al*.^45,49^.

## Results and Discussion

### Laboratory-generated aerosol

Experiments were conducted in the Georgia Tech Environmental Chamber (GTEC) facility. Typical time series for NO, NO_2_, O_3_, gas-phase naphthalene concentrations, and aerosol mass concentrations are shown in Fig. S1 for the two seed particles investigated. In both cases, NO decreased due to reaction with peroxy radicals (RO_2_), which are important radical intermediates formed from hydrocarbon oxidation, and whose fates affect the oxidation products and SOA formation^60,61^. Aerosol growth was observed shortly following the initiation of photooxidation (i.e., turning on the lights). Most of the hydrocarbon was consumed in two hours and peak aerosol mass was reached. In general, FS seeded experiments (Fig. S1B) yielded less aerosol mass compared to AS seeded experiments (Fig. S1A). Previous studies exploring the effect of iron sulfate seed on aerosol formation (e.g., α-pinene and toluene photooxidation SOA in the presence and absence of iron sulfate seed) have also reported on the decreasing effect of iron sulfate seed on SOA yield, that is less aerosol mass was formed in the presence of iron sulfate seed^50,51^.

Aerosol chemical composition was monitored using a high resolution time-of-flight aerosol mass spectrometer (HR-ToF-AMS, Aerodyne; henceforth referred to as the AMS) for all chamber experiments. The average, normalized AMS mass spectra (Fig. S2) are consistent with those reported in previous studies^62,63^. A fragmentation pattern characterized by distinct ions at *m/*z 77, 91, 105, 119, 133, 147, and 160, was observed, which is likely representative of phenylalkyl fragments^64^. Differences in AMS mass spectra between aerosol formed in the presence of AS and FS seed were observed as well (Figs S3 and S4). Elemental ratios (O:C, H:C, and N:C) of SOA were also determined using the AMS, and average aerosol carbon oxidation states (${\bar{{\rm{O}}{\rm{S}}}}_{{\rm{c}}}$ = 2 O:C–H:C)^65^ of SOA were calculated. O:C ratios and ${\overline{{\rm{O}}{\rm{S}}}}_{{\rm{c}}}$ were higher for all FS seeded SOA compared to AS seeded SOA (Table S1). This is consistent with previous laboratory studies, where the presence of iron sulfate seed resulted in the generation of more oxidized aerosol (higher O:C and ${\overline{{\rm{O}}{\rm{S}}}}_{{\rm{c}}}$) due to Fenton-type reactions^53^. Additionally, for both AS and FS seeded SOA, ${\overline{{\rm{O}}{\rm{S}}}}_{{\rm{c}}}$ followed a decreasing trend with the mass of organic aerosol formed (ΔM_o_), which is consistent with semi-volatile partitioning^66,67^ (Fig. S5). Specifically, more SOA was formed in experiments with a higher initial naphthalene concentration. With a higher aerosol mass loading, more volatile species (with a lower O:C and ${\overline{{\rm{O}}{\rm{S}}}}_{{\rm{c}}}$) will also partition into the particle phase, thus lowering the overall ${\overline{{\rm{O}}{\rm{S}}}}_{{\rm{c}}}$ of the aerosol.

### Effect of iron seed on cellular ROS/RNS production

**Figure 1:**
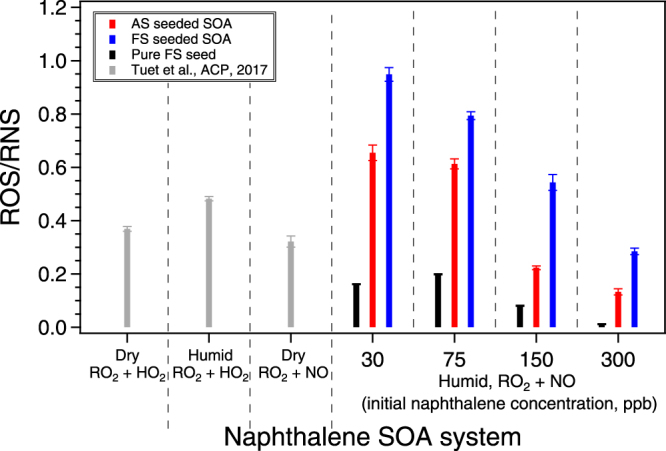
ROS/RNS produced as a result of naphthalene SOA exposure and corresponding ROS/RNS response from pure iron sulfate seed. ROS/RNS are expressed as the area under the dose-response curve (AUC). SOA were generated from the photooxidation of naphthalene in the presence of different seed particles (ammonium sulfate or iron sulfate), OH radical precursor (H_2_O_2_), and NO. Data from previous studies, where SOA were generated in the presence of ammonium sulfate seed, were included for comparison. Initial hydrocarbon concentrations for other experiments are as follows: dry, RO_2_ + HO_2_ (178 ppb); humid, RO_2_ + HO_2_ (431 ppb); and dry, RO_2_ + NO (146 ppb)^45^.

To investigate whether the presence of metal-containing seed particles affected SOA toxicity, chemical and cellular oxidant production was measured for naphthalene SOA formed in the presence of iron-containing seed vs. inorganic seed (denoted OP_seed+SOA_ or ROS/RNS_seed+SOA,_ where seed = FS or AS, where applicable). ROS/RNS production, expressed as the area under the dose-response curve (AUC) per mass of SOA (µg) in the filter extract, is shown in Fig. 1, colored by seed type. AUC was used as previous drug and aerosol studies have shown that it is the most robust dose-response metric, whose informativeness does not rely on the presence of a baseline or maximum response^20,68^. It should be noted that for all experiments, FS seeded SOA exposure resulted in higher ROS/RNS levels compared to AS seeded SOA. This observed difference can potentially be attributed to both the seed itself (FS vs. AS) and organic aerosol formed in the presence of difference seeds.

The seed effect was explored by exposing cells to pure iron sulfate seed. Exposure to both aerosolized (injected into the chamber, collected onto a filter, and extracted into media; see methods section and SI for details on filter collection and extraction) and aqueous (seed solution diluted in media) iron sulfate resulted in ROS/RNS levels that fall along the same dose-response curve (Fig. S6). This suggests that the aerosolization, collection, and extraction process does not alter the iron sulfate in a way which changes its ROS/RNS inducing ability. We then use this dose-response curve to estimate the ROS/RNS response attributable to the presence of iron sulfate alone (ROS/RNS_FS_) in SOA experiments. For each FS seeded SOA experiment, the seed mass collected onto the filter was approximated by fitting a double exponential^69^ to the seed concentration time series (in the absence of chemical reactions, prior to aerosol formation) and integrating the fitted function over the filter collection period (Fig. S7). The corresponding ROS/RNS_FS_ response as a result of exposure to this seed mass was then calculated using the iron sulfate dose-response curve (Fig. S6). These calculations were only performed for FS seeded SOA as exposure to ammonium sulfate seed has previously been shown to induce negligible ROS/RNS response at similar seed mass concentrations^49^. The ROS/RNS_FS_ response based on the determined iron sulfate seed mass accounted for about 2–12% of the measured ROS/RNS_FS+SOA_ response. It should be noted that these estimated contributions are only simple approximations to provide perspective as concentration addition may not apply for cellular responses. Nevertheless, these results are interesting as pure iron sulfate seed induced relatively low ROS/RNS production compared to that induced by the collected samples (i.e., ROS/RNS_FS_ $\ll$ ROS/RNS_FS+SOA_). This suggests that the measured ROS/RNS_FS+SOA_ response may be predominantly attributed to organic components. These results confirm the importance of organic species to aerosol health effects, and previous studies on ROS/RNS produced as a result of aerosol exposure have also found significant correlations between the concentration of water soluble organic carbon (WSOC) and ROS/RNS response^27,70–72^.

**Figure 2:**
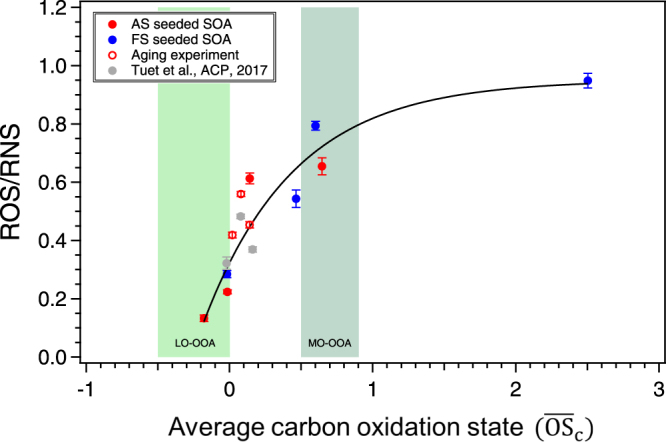
Exponential trend between ROS/RNS levels and average carbon oxidation state (${\overline{{\rm{O}}{\rm{S}}}}_{{\rm{c}}}$) for naphthalene photooxidation SOA generated in the presence of different seed particles (ammonium sulfate or iron sulfate), OH radical precursor (H_2_O_2_), and NO. ROS/RNS production are expressed as the area under the dose-response curve (AUC). Error bars were determined using the methodolgy outlined in Tuet *et al*.^20^. Data from previous studies were included for comparison^45^. ${\overline{{\rm{O}}{\rm{S}}}}_{{\rm{c}}}$ ranges for less oxidized oxygenated organic aerosol (LO-OOA) and more oxidized OOA (MO-OOA) are shaded for context^65^.

The degree of oxidation is a parameter of interest for organic aerosol, as atmospheric photochemical aging occurs over an aerosol’s lifetime, yielding more oxidized species and aerosol with a higher ${\overline{{\rm{O}}{\rm{S}}}}_{{\rm{c}}}$ ^65^. The observed difference in ROS/RNS levels between AS and FS seeded SOA is likely an effect of the degree of oxidation, where the presence of iron serves to increase the oxidation of species via Fenton-like reactions (Table S1)^51,52^. In fact, a positive exponentially decreasing trend was observed between ROS/RNS levels and ${\overline{{\rm{O}}{\rm{S}}}}_{{\rm{c}}}$ of aerosol for all experiments (Fig. 2). These results are consistent with our previous study on the ROS/RNS levels of SOA generated from various precursors, where a significant positive correlation was observed between ROS/RNS and ${\overline{{\rm{O}}{\rm{S}}}}_{{\rm{c}}}$ ^49^. Results from this study therefore further support the idea that more oxidized products are likely better oxidizing agents which can induce higher levels of ROS/RNS. In addition, the observed trend suggests that different seed types do not affect the ROS/RNS response as both AS and FS seeded SOA fall on the same ROS/RNS vs ${\overline{{\rm{O}}{\rm{S}}}}_{{\rm{c}}}$ curve.

**Figure 3:**
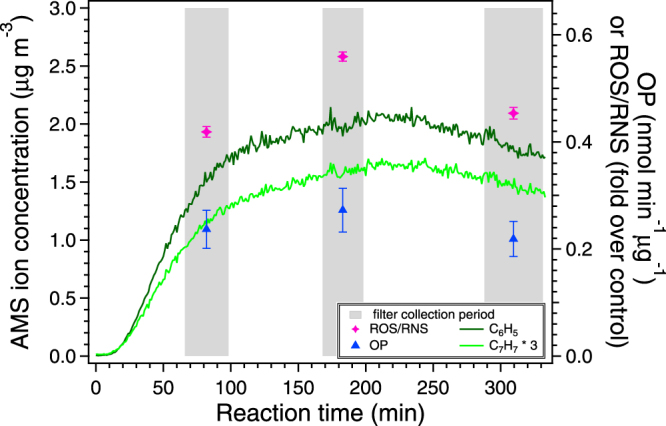
Intrinsic OP and ROS/RNS levels for naphthalene photooxidation SOA collected over the course of a single experiment (Expt. 5). Time series for AMS *m/*z 77 and 91, which are likely phenyl and benzyl ions, are also shown. SOA was generated in a humid chamber in the presence of ammonium sulfate, OH radical precursor (H_2_O_2_), and NO. Error bars represent a 15% coefficient of variation for OP^21^. ROS/RNS levels are expressed as the area under the dose-response curve (AUC) with error bars calculated following the methodology described in Tuet *et al*.^20^.

It is also interesting to note that the ROS/RNS levels for filter samples collected over the course of a single experiment (Expt. 5) roughly follow the time series for aromatic phenyl and benzyl ions measured by the AMS (*m/z* 77 and 91, respectively, Fig. 3). Previous studies comparing cellular inflammantory responses from naphthalene and *m*-xylene SOA have suggested that aromatic-retaining products may have significant health implications^45,49^. While results from this study are not sufficient to conclude causation, these observations along with findings from previous studies on the importance of humic-like substances (HULIS)^28,30,31^ should inspire future studies to focus on assessing the health implications of aromatic SOA and determine whether the presence of aromaticity directly induces adverse outcomes.

The ROS/RNS levels induced by naphthalene SOA generated under different formation conditions (e.g., RH, peroxy radical fate, OH source) have been measured in our previous study^49^ and are also shown in Fig. 1 for comparison. In both the previous and this study, the same cellular assay and analysis method was utilized. However, comparing ROS/RNS levels directly between these two studies may not be applicable as there are several differences between SOA formation condition (e.g., different initial naphthalene concentrations, different relative humidities, and different OH radical precursors)^49^. It is interesting to note that the exponential relationship between ROS/RNS and ${\overline{{\rm{O}}{\rm{S}}}}_{{\rm{c}}}$ holds for all naphthalene SOA generated under different formation conditions (Fig. 2).

### Effect of iron seed on OP

**Figure 4:**
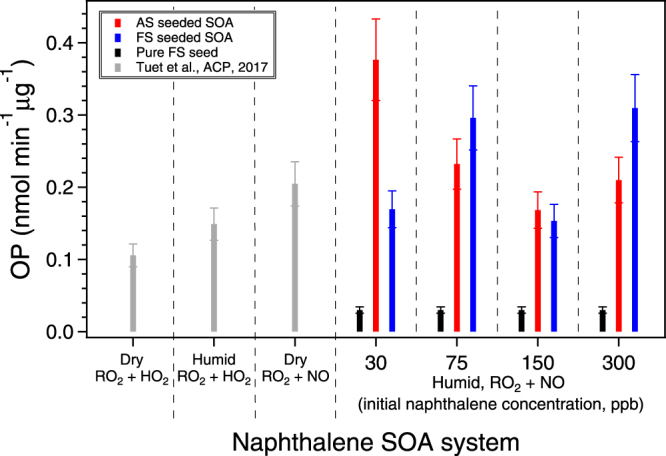
Intrinsic OP for SOA generated from the photooxidation of naphthalene under various conditions and pure iron sulfate seed. SOA from this study was generated in a humid chamber in the presence of different seed particles (ammonium sulfate or iron sulfate), OH radical precursor (H_2_O_2_), and NO. Data from previous studies, where SOA were generated in the presence of ammonium sulfate seed, were included for comparison. Initial hydrocarbon concentrations for other experiments are as follows: dry, RO_2_ + HO_2_ (178 ppb); humid, RO_2_ + HO_2_ (431 ppb); and dry, RO_2_ + NO (146 ppb)^45^.

Intrinsic OP values (per µg) for naphthalene SOA (OP_seed+SOA_) and pure iron sulfate seed (OP_FS_) are shown in Fig. 4, colored by seed type. For each FS seeded SOA experiment, the contribution of seed alone to the overall OP_FS+SOA_ level is relatively low (<20%), which parallels that observed for the ROS/RNS response. It should be noted that DTT does not respond significantly to iron, and the low OP_FS_ is consistent with previous studies, where a low DTT reactivity by iron was observed^54^. Previous studies have shown that AS alone is not redox active, that is OP_AS_ is equivalent to the response of a blank filter within experimental error^45^. It is therefore also interesting to note that OP_FS+SOA_ is not always higher than OP_AS+SOA,_ suggesting that the presence of iron seed does not always induce an additive effect. Further studies should explore various effect models for OP to investigate additivity.

Overall, there are no apparent trends for the OP values obtained for SOA generated using different initial naphthalene concentrations (hence different organic aerosol mass loadings and ${\overline{{\rm{O}}{\rm{S}}}}_{{\rm{c}}}$) or in the presence of different seed types. Furthermore, there was no observable relationship between OP and ${\overline{{\rm{O}}{\rm{S}}}}_{{\rm{c}}}$ (Fig. S8). While these results are in contrast to trends observed for ROS/RNS levels, they are consistent with previous studies on the DTT activities of different SOA systems and various ambient PM subtypes^23,45,73,74^. Tuet *et al*.^45^ previously measured the intrinsic OP of different SOA systems (including naphthalene SOA) and found that while different SOA precursors and formation conditions produced SOA of differing ${\overline{{\rm{O}}{\rm{S}}}}_{{\rm{c}}}$, there was no apparent relation between OP and ${\overline{{\rm{O}}{\rm{S}}}}_{{\rm{c}}}$. The study also showed that for both laboratory-generated SOA and different organic aerosol subtypes^23,73,74^ resolved from ambient data, a higher ${\overline{{\rm{O}}{\rm{S}}}}_{{\rm{c}}}$ did not correspond to a higher OP. It should be noted that these results may be complicated by mixture effects and/or dependent on the PM subtype, as previous studies have found that oxidation of quinones, diesel exhaust, or freshly emitted trash-burning aerosol enhances their redox activity^28,75,76^. Nevertheless, results from this study may further highlight the differences between chemical and cellular assays. More specifically, it was suggested in a previous study by Tuet *et al*. that chemical assays, such as DTT, may only be sensitive to larger differences (i.e., different SOA precursors rather than different SOA formation conditions), while cellular assays are sensitive to differences arising from different SOA formation conditions as well as SOA precursor^49^. The lack of correlation between OP and ${\overline{{\rm{O}}{\rm{S}}}}_{{\rm{c}}}$ in this study may therefore be a result of the fact that all SOA in this study were generated from the same precursor (i.e., naphthalene) under the same formation condition (same RH and OH source). The specific oxidants (exogenous vs. endogenous) measured by each assay may be another potential explanation for the differences observed. DTT is primarily a measure of endogenous oxidant production as it is sensitive to redox-active species capable of interacting with anti-oxidants and less sensitive to the oxidants themselves (exogenous oxidants, e.g., H_2_O_2_). The cellular ROS/RNS assay also predominantly measures post-exposure endogenous oxidant production since extracellular ROS/RNS probe is removed after the probe incubation time^20^. However, while the cellular assay may not directly measure exogenous oxidants, these species can interact with cells and induce pathways that may produce ROS/RNS. Therefore, the cellular assay may contain contributions from both endogenous and exogenous oxidants, while the DTT assay is largely a measure of only endogenous oxidants.

### Relationship between photochemical aging of aerosol and oxidant production

As the laboratory experiment progressed, OH exposure of aerosol and ${\overline{{\rm{O}}{\rm{S}}}}_{{\rm{c}}}$ increased as a result of increased photochemial aging. To investigate whether the effects of photochemical aging are comparable to those observed for SOA of different ${\overline{{\rm{O}}{\rm{S}}}}_{{\rm{c}}}$ (a proxy for aging), multiple filter samples were collected over the course of a single experiment (Table 1, repeat of Expt. 5). It should be noted that this aging experiment is an exact repeat of the previous experiment (Expt. 5), with the exception of a longer experimental time and multiple filter sample collections to explore changes in ${\overline{{\rm{O}}{\rm{S}}}}_{{\rm{c}}}$ associated with photochemical aging. The ROS/RNS levels and OP for these samples are shown in Fig. 3. The OP for these three samples are the same within uncertainty, consistent with the hypothesis that the DTT assay may only be sensitive to larger differences (such as precursor identity). On the other hand, the ROS/RNS response followed the same trend as that of ${\overline{{\rm{O}}{\rm{S}}}}_{{\rm{c}}}$. The ROS/RNS response induced by these samples and the ${\overline{{\rm{O}}{\rm{S}}}}_{{\rm{c}}}$ calculated for each collection period are also shown in Fig. 2 (opened markers) for comparison. These values fall within the exponential trend observed between ROS/RNS and ${\overline{{\rm{O}}{\rm{S}}}}_{{\rm{c}}}$ for SOA generated from different initial hydrocarbon concentrations. This suggests that the proxy for aging (${\overline{{\rm{O}}{\rm{S}}}}_{{\rm{c}}}$) investigated in this study may be used to understand the potential health implications of aged particles for SOA from a single pure compound.

**Table 1:** Experimental conditions.

Experiment	Hydrocarbon	Seed	Relative humidity	[HC]_0_	[NO]_0_	[SOA]^c^
			(%)	(ppb)	(ppb)	(µg m^−3^)
1	naphthalene	AS^a^	51%	32	315	11.7
2	naphthalene	FS^b^	50%	32	303	7.28
3	naphthalene	AS^a^	49%	92	368	66.7
4	naphthalene	FS^b^	48%	84	214	24.0
5^d^	naphthalene	AS^a^	54%	186	344	187
6	naphthalene	FS^b^	52%	182	321	149
7	naphthalene	AS^a^	53%	342	320	348
8	naphthalene	FS^b^	51%	331	295	369

^a^Ammonium sulfate seed (15 mM (NH_4_)_2_SO_4_); ^b^Iron sulfate seed (15 mM FeSO_4_); ^c^Average SOA concentration in the chamber during filter collection; ^d^Experiment was repeated and multiple filters were collected over the course of the experiment to investigate the effects of photochemical aging.

These observations have significant implications for future health studies as atmospheric aging leads to increases in aerosol oxidation^33,63^, which may affect cellular responses. This is important as aerosol have an atmospheric lifetime of about a week, over which these aging processes can occur. If the observed relationship between cellular ROS/RNS response and ${\overline{{\rm{O}}{\rm{S}}}}_{{\rm{c}}}$ holds for other SOA systems, as well as ambient mixtures, these results may lead to ROS/RNS predictions based on more accessible bulk aerosol properties that are readily measured by the AMS. These approximations would not require the additional processing (e.g., filter collection and extraction) that actual ROS/RNS measurements entail. As an example, the ${\overline{{\rm{O}}{\rm{S}}}}_{{\rm{c}}}$ ranges for various organic aerosol subtypes resolved from ambient data world-wide, specifically less-oxidized oxygenated organic aerosol (LO-OOA) and more-oxidized OOA (MO-OOA), have been measured previously and are shaded in Fig. 2 to provide context^65,73,74^. ROS/RNS levels measured in this study span the shaded regions, and the observed exponential trend suggests that exposure to MO-OOA would likely induce more ROS/RNS production compared to LO-OOA. This may have important implications as studies have shown that ambient organic aerosol from different sources converge towards MO-OOA as they age^33,35^, and MO-OOA has widespread contributions to organic aerosol in both rural and urban locations across different seasons^33,35,73,74^. Additional studies are required to establish whether the ROS/RNS and ${\overline{{\rm{O}}{\rm{S}}}}_{{\rm{c}}}$ relationship holds for different aerosol systems as previous studies have shown that SOA generated from different precursors induce different cellular inflammatory responses^49^.

### Implications

The intracellular ROS/RNS production and water-soluble OP were measured for naphthalene photooxidation SOA formed under humid conditions in the presence of metal-containing and inorganic seed. Experiments were conducted using different initial hydrocarbon concentrations to generate aerosol of differing mass loadings and degrees of oxidation. Multiple filters were also collected from a single experiment to obtain aerosol of different photochemical age. Cellular assay results show that exposure to FS seeded aerosol resulted in higher levels of ROS/RNS production compared to AS seeded aerosol. Furthermore, the ROS/RNS response may be largely attributed to the organic components rather than the metals portion. This has important implications for future studies as organic aerosol constitute a large fraction of ambient fine PM^32,33^. However, it should be noted that possible synergistic and/or antagonistic metal-organic interactions were not explored and only one metal species and volatile organic compound (VOC) were investigated in this study. Further studies are necessary to determine how metals and organics interact with each other and in the context of biologically-relevant species (e.g. proteins, sugars, and lipids present in the alveolar fluid). These interactions between co-exposed species may increase or decrease the overall cellular response^77–79^, and a thorough understanding of these dynamics are necessary to evaluate the health implications of ambient aerosol. Results from this study also highlight the differences between chemical and cellular assays. There were no obvious trends between OP values and aerosol bulk composition meaured by the AMS, suggesting that the DTT assay may only be sensitive to large differences, such as that arising from different SOA precursors. The lack of correlation between OP and ${\overline{{\rm{O}}{\rm{S}}}}_{{\rm{c}}}$ is consistent with previous DTT studies, where a higher ${\overline{{\rm{O}}{\rm{S}}}}_{{\rm{c}}}$ did not correspond to a higher OP^23,73,74^.

An exponential trend was also observed between ROS/RNS levels and ${\overline{{\rm{O}}{\rm{S}}}}_{{\rm{c}}}$ for all naphthalene photooxidation SOA, including those formed in the presence of different seed particles (AS and FS), those formed under different formation conditions (dry vs. humid, RO_2_ + HO_2_ vs. RO_2_ + NO), and those collected at different times over the course of a single experiment (different degrees of photochemical aging). There are several important implications arising from this trend. For one, the trend implies that there is negligible seed effect with respect to ROS/RNS produced as a result of SOA exposure. The aerosol formed in all experiments fall on the same ROS/RNS vs. ${\overline{{\rm{O}}{\rm{S}}}}_{{\rm{c}}}$ curve regardless of whether AS or FS seed was used. Hence, the observed difference between AS and FS seeded SOA (where all FS seeded SOA induced more ROS/RNS production) is likely an effect of differences in the degree of aerosol oxidation resulting from increased free radical production via Fenton-like reactions. The aerosol collected at multiple time points over the course of a single experiment (prolonged aging experiment) yield results that fall along this curve as well, which suggests that results obtained using ${\overline{{\rm{O}}{\rm{S}}}}_{{\rm{c}}}$ (a proxy for aging) may be generalized for photochemical atmospheric aging for this parent VOC and specific metal. Further studies are still required to establish whether the observed relationship between ROS/RNS and ${\overline{{\rm{O}}{\rm{S}}}}_{{\rm{c}}}$ holds for other aerosol systems, as only naphthalene photooxidation SOA was investigated in this study. Ambient aerosol are complex mixtures formed from multiple precursors and containing a variety of metallic species. These mixtures have not been considered in this study, and results may be different due to synergistic and antagonistic mixture effects that have yet to be explored. However, if measures of bulk aerosol oxidation state (i.e ${\overline{{\rm{O}}{\rm{S}}}}_{{\rm{c}}}$) are validated with more aerosol systems to be used as a proxy for cellular ROS/RNS produced upon aerosol exposure, then the ability to perform more bulk aerosol measurements may lead to ROS/RNS predictions in the absence of cellular measurements.

## Methods

### Naphthalene aerosol generation

Naphthalene photooxidation SOA (naphthalene + hydroxyl (OH) radical) was generated under humid conditions in the presence of NO in the Georgia Tech Environmental Chamber (GTEC) facility. Briefly, the facility consists of two 12 m^3^ Teflon^TM^ chambers suspended inside a temperature-controlled enclosure surrounded by black lights (Sylvania 24922) and natural sunlight fluorescent lamps (Sylvania 24477)^80^. Each chamber is equipped with multiple sampling ports for reagent introduction and various gas- and aerosol-phase measurements. NO_2_, NO_x_, and O_3_ were monitored using a cavity-attenuated phase shift (CAPS) NO_2_ monitor (Aerodyne), a chemiluminescence NO_x_ monitor (Teledyne 200EU), and an O_3_ analyzer (Teledyne T400), respectively. Hydrocarbon concentration was monitored using a gas chromatography flame ionization detector (GC-FID, Agilent 7890 A) and hydroxyl radical concentration was calculated from the hydrocarbon decay. Aerosol volume concentrations and size distributions as well as bulk aerosol compositions were measured using a scanning mobility particle sizer (SMPS, TSI) and a high resolution time-of-flight aerosol mass spectrometer (HR-ToF-AMS, Aerodyne; henceforth referred to as the AMS), respectively^81^. AMS data were analyzed using data analysis toolkits SQUIRREL (v. 1.57) and PIKA (v. 1.16), while elemental ratios (O:C, H:C, and N:C) were determined using the method outlined in Canagaratna *et al*.^82^ O:C and H:C ratios were then used to calculate the average carbon oxidation state (${\overline{{\rm{O}}{\rm{S}}}}_{{\rm{c}}}$)^65^. Finally, temperature and relative humidity (RH) were monitored using a hydro-thermometer (Vaisala HMP110).

Experimental conditions, given in Table 1, were designed to probe the effects of metal seed and aerosol chemical composition on OP and intracellular ROS/RNS production. All experiments were performed at ~25 °C under humid conditions (RH ~50%). Prior to each experiment, chambers were flushed with pure air and humidified using a bubbler filled with deionized (DI) water. Once the desired humidity was reached, seed aerosol was injected by atomizing seed solution (15 mM (NH_4_)_2_SO_4_ for ammonium sulfate (AS) experiments and 15 mM FeSO_4_ for iron sulfate (FS) experiments (Sigma Aldrich)) until the seed concentration inside the chamber was approximately 30 µg m^−3^. Naphthalene was then injected by passing pure air at 5 L min^−1^ over solid naphthalene flakes (99%, Sigma Aldrich)^83^. NO (500 ppm, Matheson) and OH precursor (H_2_O_2_, 50% aqueous solution, Sigma Aldrich) were injected afterwards to attain an initial NO concentration of 300 ppb and an H_2_O_2_ concentration of 3 ppm, which yielded OH concentrations on the order of 10^6^–10^7^ molec cm^−3^. Once all reagent concentrations stabilized, UV lights were switched on to initiate photooxidation.

### Aerosol collection and extraction

Aerosol samples were collected at peak growth onto 47 mm Teflon^TM^ filters (0.45 µm pore size, Pall Laboratory) for 1.6 hrs at a flow rate of 29 L min^−1^. The total mass collected on each filter was determined by integrating time-dependent SMPS volume concentrations over the filter collection period and multiplying the integrated value by the total volume of air collected. A density of 1 g cm^−3^ was assumed to facilitate comparison between studies, as SOA density varies with precursor identity and formation condition^83–88^. Background filters containing only seed (AS or FS), OH precursor (H_2_O_2_), and NO at experimental conditions were also collected to account for potential H_2_O_2_ uptake onto seed particles since this may affect oxidative potential and ROS/RNS measurements. After collection, filters were placed in sterile petri dishes, sealed with Parafilm M^®^, and stored at −20 °C until extraction and analysis^21^.

Collected filter samples were extracted following the procedure outlined in Fang *et al*.^22^ with modifications for cellular exposure described in Tuet *et al*.^20^. Briefly, filters were submerged in extraction media (DI water for OP and cell culture media (RPMI-1640) for ROS/RNS) and sonicated for two 30 min intervals using an Ultrasonic Cleanser (VWR International). Post-sonication, sample extracts were filtered using a 0.45 µm polytetrafluoroethylene (PTFE) syringe filter (Fisherbrand^™^) to remove insoluble material^21^ and extracts for cellular exposure were supplemented with 10% fetal bovine serum (FBS).

### Oxidative potential

The intrinsic water soluble oxidative potential as measured by DTT (OP) of naphthalene aerosol, method blanks, and positive controls (9,10-phenanthraquinone) were determined using a semi-automated DTT system, described in detail in Fang *et al*.^21^. Briefly, the method consisted of three major steps: (1) oxidation of DTT by redox-active species in the extract, (2) reaction of remaining DTT with 5,5-dithio-bis-(2-nitrobenzoic acid) (DTNB) to form 2-nitro-5-mercaptobenzoic acid (TNB), and (3) measurement of TNB at 412 nm.

### Intracellular ROS/RNS measurement

Murine alveolar macrophages (MH-S, ATCC^®^CRL-2019^™^) were cultured in RPMI-1640 media supplemented with 10% FBS, 1% penicillin-streptomycin, and 50 µM β-mercaptoethanol (BME) at 37 °C and 5% CO_2_. ROS/RNS were detected using the assay described in Tuet *et al*.^20^. The assay consisted of five steps: pre-treatment of 96-well plates with 10% FBS in phosphate buffered saline (PBS), (2) seeding of cells at 2 × 10^4^ cells well^−1^, (3) incubation of cells with ROS/RNS probe (10 µM, carboxy-H_2_DCFDA, Molecular Probes C-400), (4) exposure of cells to samples and controls for 24 hrs, and (5) detection of ROS/RNS using a microplate reader (BioTek Synergy H4, ex: 485 nm, em: 525 nm). Positive controls included bacterial cell wall component, lipopolysaccharide (LPS, 1 µg mL^−1^), H_2_O_2_ (100 µM), and reference filter extract (10 filter punches mL^−1^, 1 per filter sample, from various ambient filters collected at the Georgia Tech site^20^; negative controls included blank filter extract and control cells (probe-treated cells exposed to media only, no stimulants).

For each filter sample, intracellular ROS/RNS production was measured over ten doses to fully capture dose-response relationships (Fig. S9). At each dose, ROS/RNS levels were normalized to basal ROS/RNS production^89^ (i.e. ROS/RNS produced from probe-treated control cells) and corrected for changes in relative cellular metabolic activity^90^ (measured using MTT, 3-(4,5-dimethylthiazol-2-yl)-2,5-diphenyltetrazolium bromide, assay) (Biotium) prior to fitting dose-response curves. Area under the dose-response curve (AUC) was then used to represent ROS/RNS for comparison to chemical oxidative potential as AUC is the most robust metric for comparing different PM samples^20^.

### Cellular metabolic activity

MTT was used to assess cellular metabolic activity post-exposure. Sample extracts were removed after the exposure period (24 hrs), replaced with media containing MTT, and returned to the incubator for 4 hrs. Dimethyl sulfoxide was then added to solubilize the insoluble purple salt formed from the reduction of the tetrazolium dye and the absorbance at 570 nm was measured using a microplate reader (BioTek Synergy H4).

### Data availability

Data are available upon request to the corresponding author (ng@chbe.gatech.edu).

## Electronic supplementary material

Supplementary Information
