# Supplementary material for: Chemical and cellular oxidant production induced by naphthalene secondary organic aerosol (SOA): effect of redox-active metals and photochemical aging
Source: Sci Rep. 2017 Nov 9;7:15157. doi: 10.1038/s41598-017-15071-8 (PMC5680346; doi:10.1038/s41598-017-15071-8)
Supplement: Supplementary file 1 — Supplementary Information [file 41598_2017_15071_MOESM1_ESM.pdf]

Supplementary information for:

Chemical and cellular oxidant production induced by naphthalene secondary organic aerosol (SOA): effect of redox-active metals and photochemical aging

Wing Y. Tuet[1], Yunle Chen[2], Shierly Fok[1], Dong Gao[3], Rodney J. Weber[4], Julie A. Champion[1], Nga L. Ng[1,4]*

[1]School of Chemical and Biomolecular Engineering, Georgia Institute of Technology, Atlanta, GA

[2]School of Materials Science and Engineering, Georgia Institute of Technology, Atlanta, GA

[3]School of Civil and Environmental Engineering, Georgia Institute of Technology, Atlanta, GA

[4]School of Earth and Atmospheric Sciences, Georgia Institute of Technology, Atlanta, GA

Corresponding Author

[*]email: ng@chbe.gatech.edu

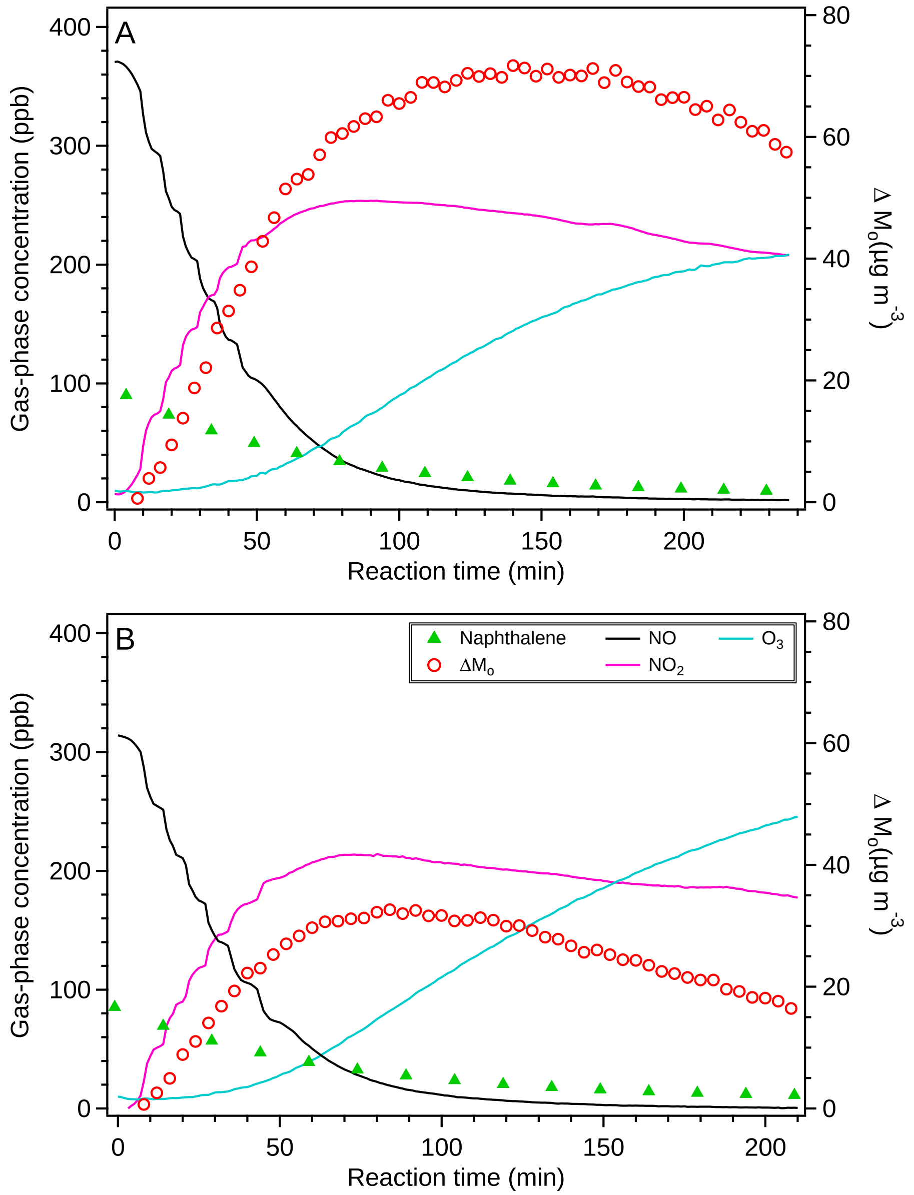

Figure S1. Typical reaction profile for a chamber experiment under humid conditions in the presence of NO using A) ammonium sulfate seed particles (Expt. 3) and B) iron sulfate seed particles (Expt. 4). Naphthalene and NO concentrations were monitored using a GC-FID and chemiluminescence NO_x monitor, respectively. Aerosol mass concentrations were determined using SMPS volume concentration and assuming an aerosol density of 1 g cm^{-3}. It should be noted that aerosol mass concentrations have not been corrected for particle wall loss.

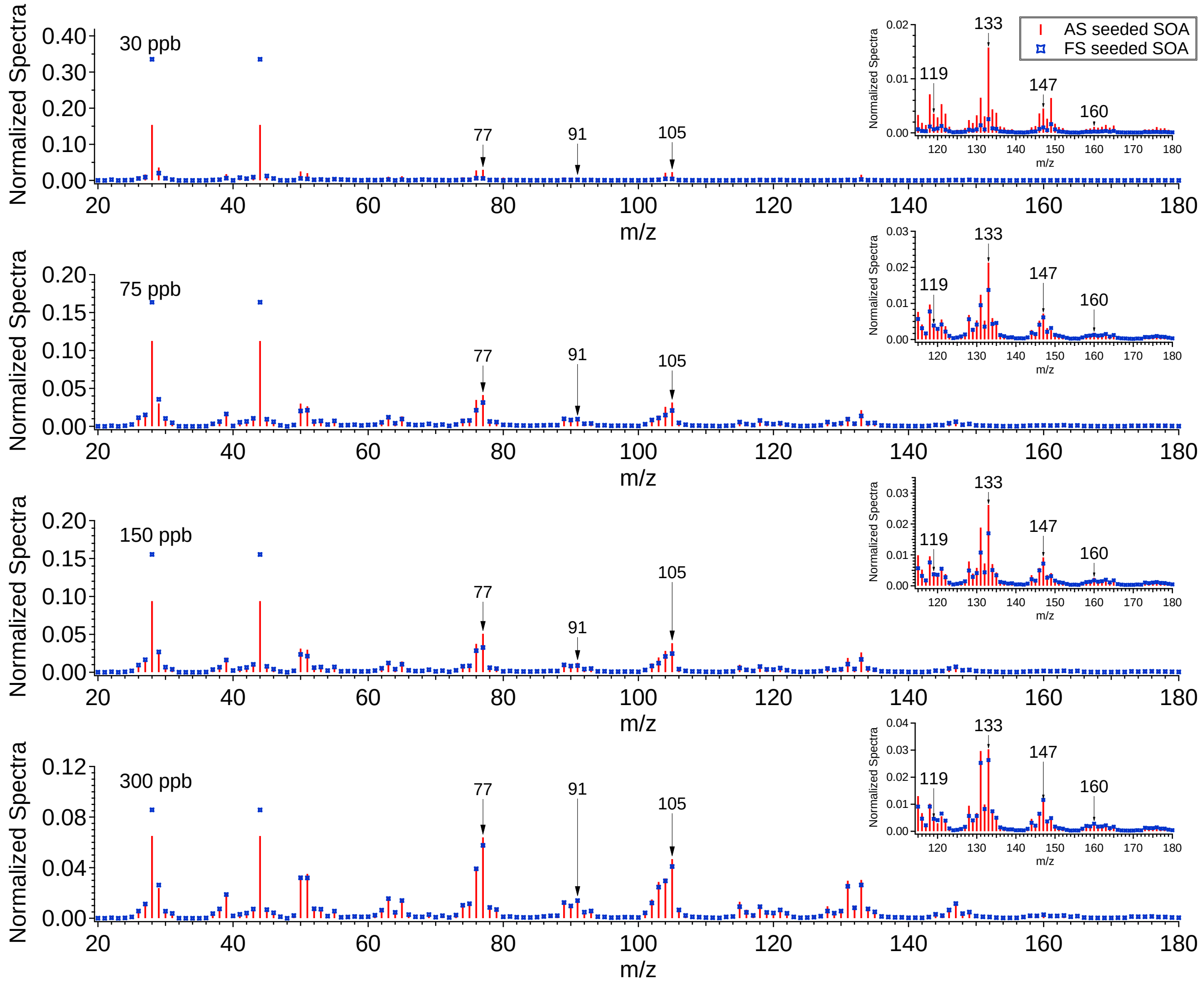

Figure S2. Aerosol mass spectra of SOA formed from the photooxidation of naphthalene under humid conditions in the presence of NO using various seed (**red bars**: ammonium sulfate; **blue markers**: iron sulfate). Each row represents a different initial naphthalene concentration (30, 75, 150, and 300 ppb). Characteristics fragments are labeled. Ions greater than *m/z* 120 are shown in the inset of each mass spectrum.

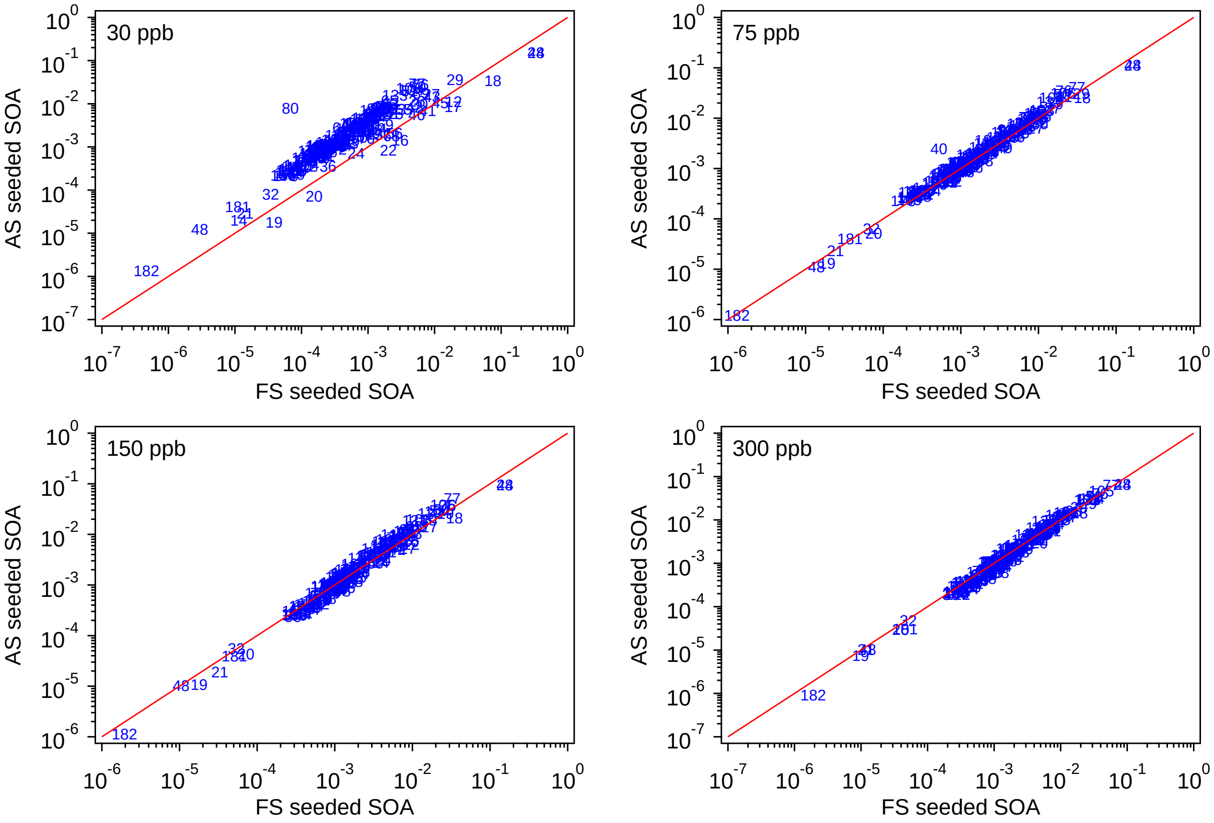

Figure S3. Comparison between mass spectra of SOA formed from the photooxidation of naphthalene under humid conditions in the presence of NO using various seeds. A 1:1 line is shown for reference.

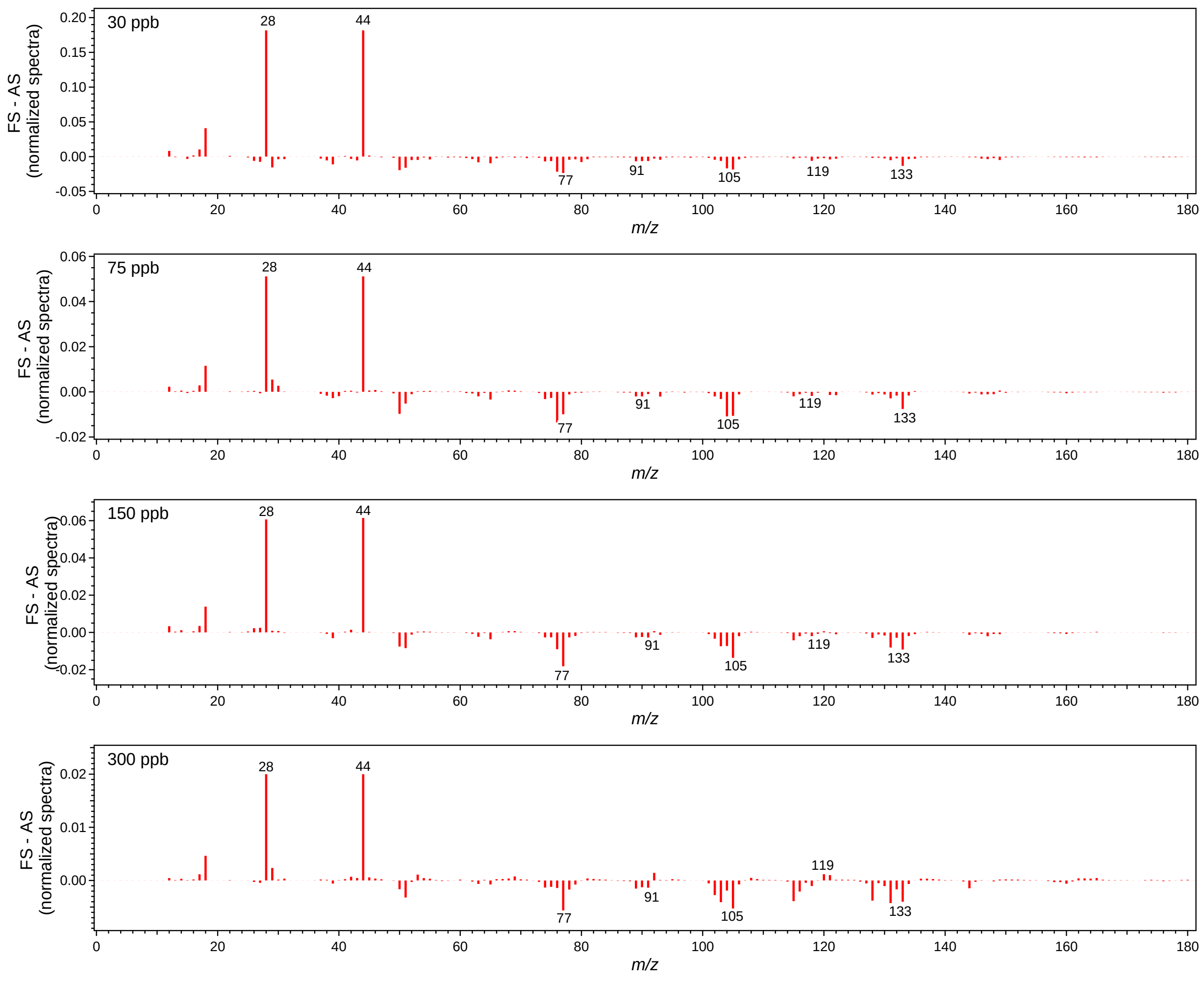

Figure S4. Difference (FS seeded SOA – AS seeded SOA) between normalized mass spectra of SOA formed from the photooxidation of naphthalene under humid conditions in the presence of NO using various seeds.

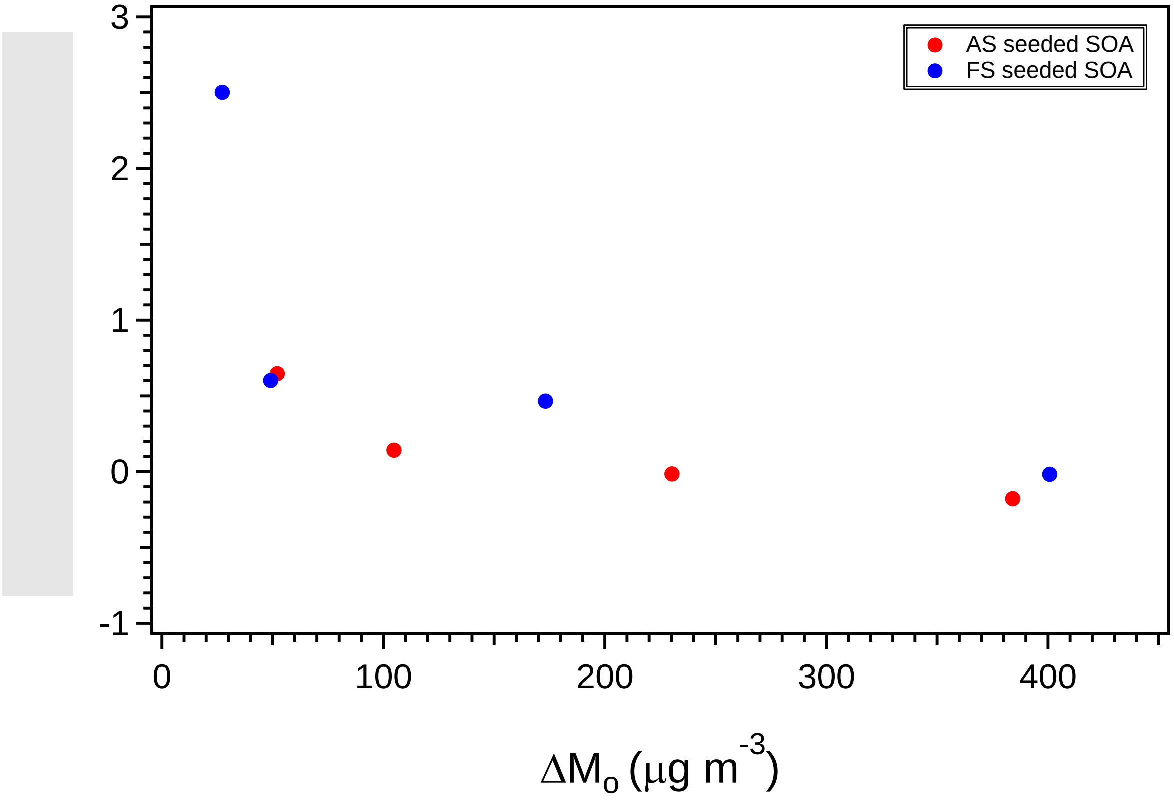

Figure S5. Average carbon oxidation state ($\overline{OS}_c$) for naphthalene SOA spanning a range of organic mass loading (ΔM_o). SOA from this study was generated in a humid chamber in the presence of different seed particles (**ammonium sulfate** or **iron sulfate**), OH radical precursor (H_2O_2), and NO.

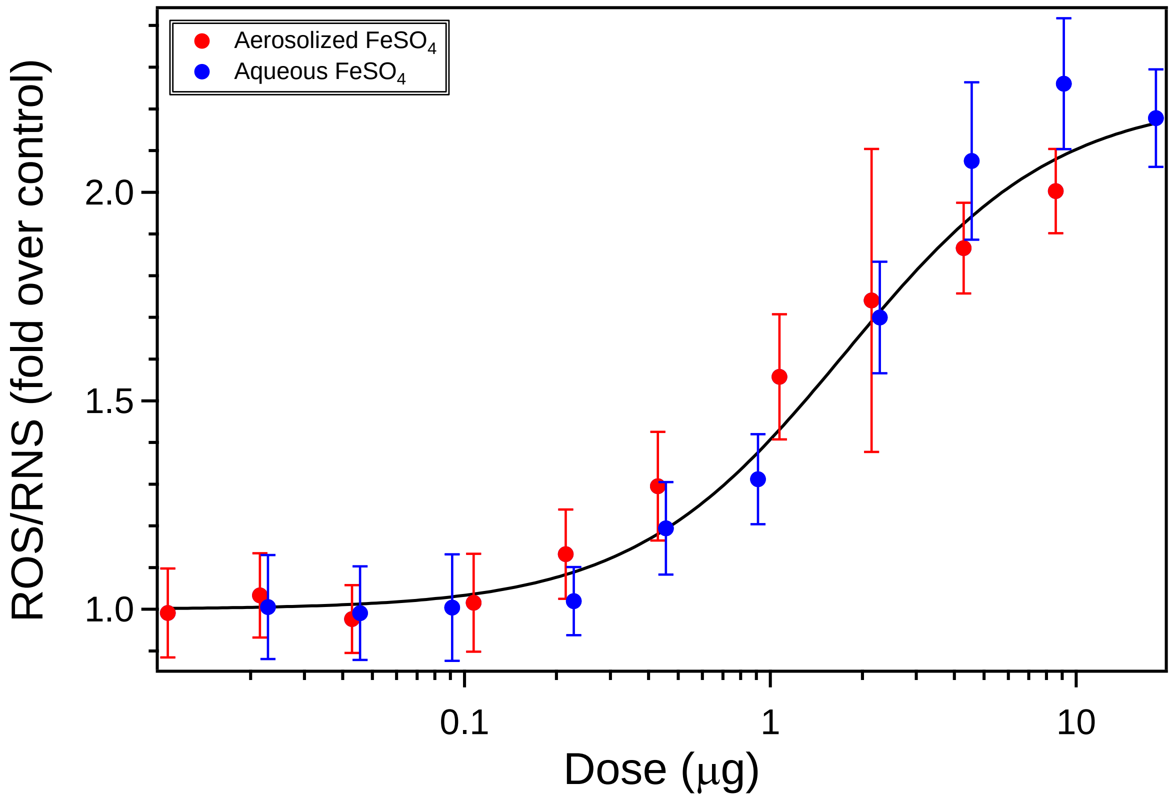

Figure S6. ROS/RNS produced as a result of exposure to $FeSO_4$ (**red**: aerosolized into the chamber at experimential concentration, collected onto a filter, and extracted into media; **blue**: aqueous seed solution diluted in media). ROS/RNS is expressed as a fold increase over probe-treated control cells incubated with stimulant-free media. Data shown are means ± standard error of triplicate exposure experiments.

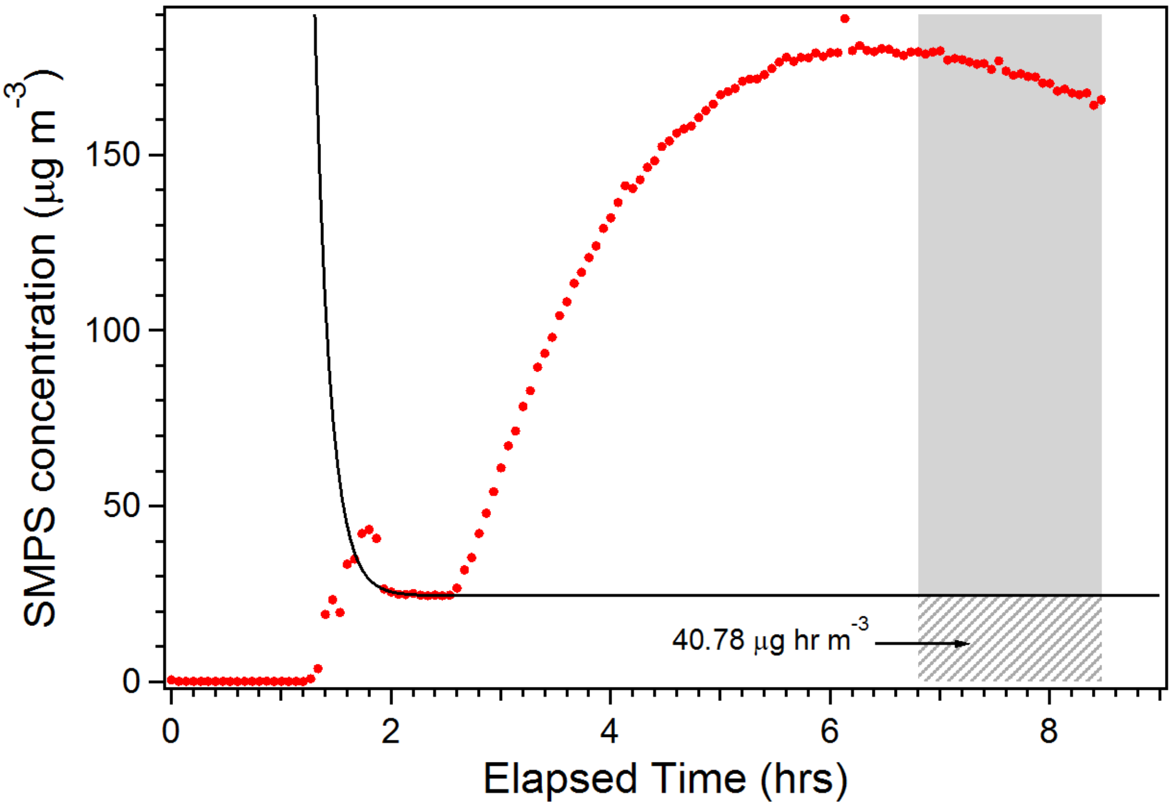

Figure S7. Method for estimating the mass of seed collected onto each filter (Expt. 6). A double exponential was used to characterize seed particle wall loss (using seed concentrations obtained from the SMPS as a function of time). The fitted seed concentration as a function of time was then integrated over the filter collection period (shown as the shaded region). To obtain the total mass of seed collected, the integral (40.78 µg m^{-3}) was multiplied by the volumetric flow rate (1.72 m^3 hr^{-1}, for an estimated total seed mass of 70.14 µg on the filter for this experiment).

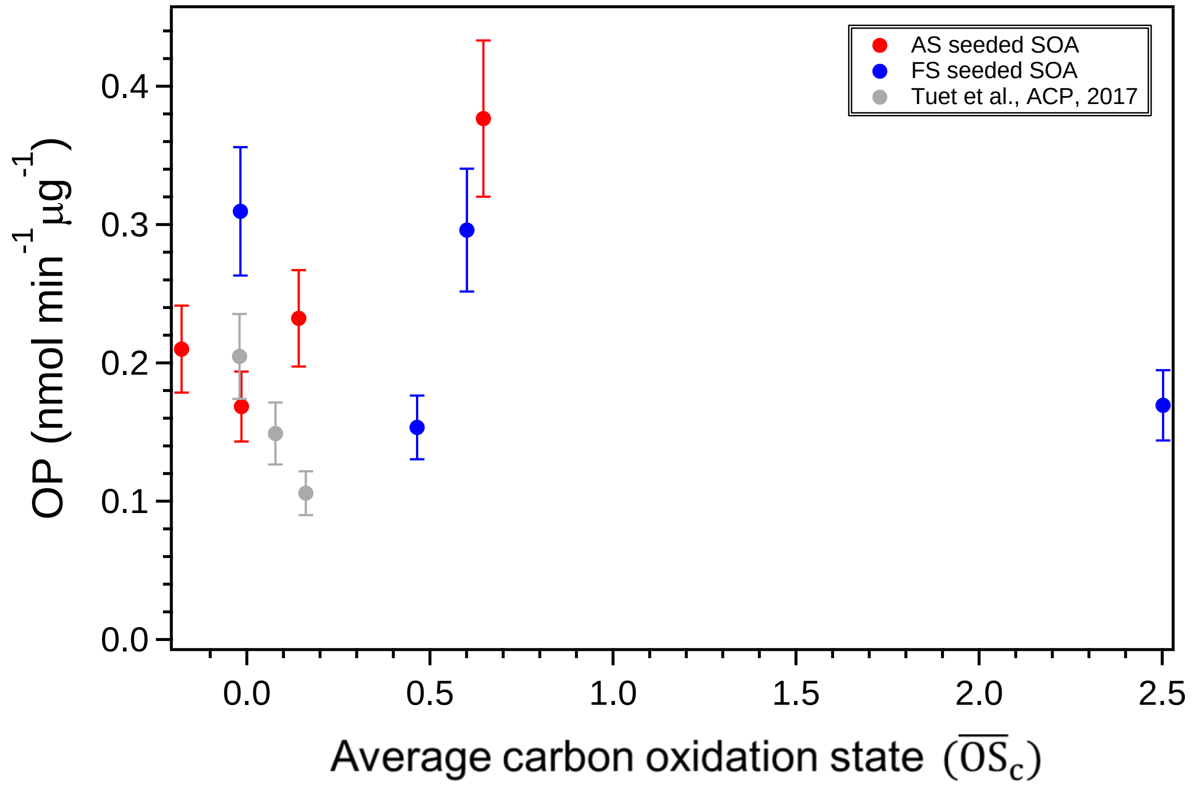

Figure S8. Intrinsic OP for naphthalene SOA spanning a range of average carbon oxidation states ($\overline{OS}_c$). SOA from this study was generated in a humid chamber in the presence of different seed particles (**ammonium sulfate** or **iron sulfate**), OH radical precursor (H_2O_2), and NO. Error bars represent a 15% coefficient of variation.[1] Data from previous studies were included for comparison.[2]

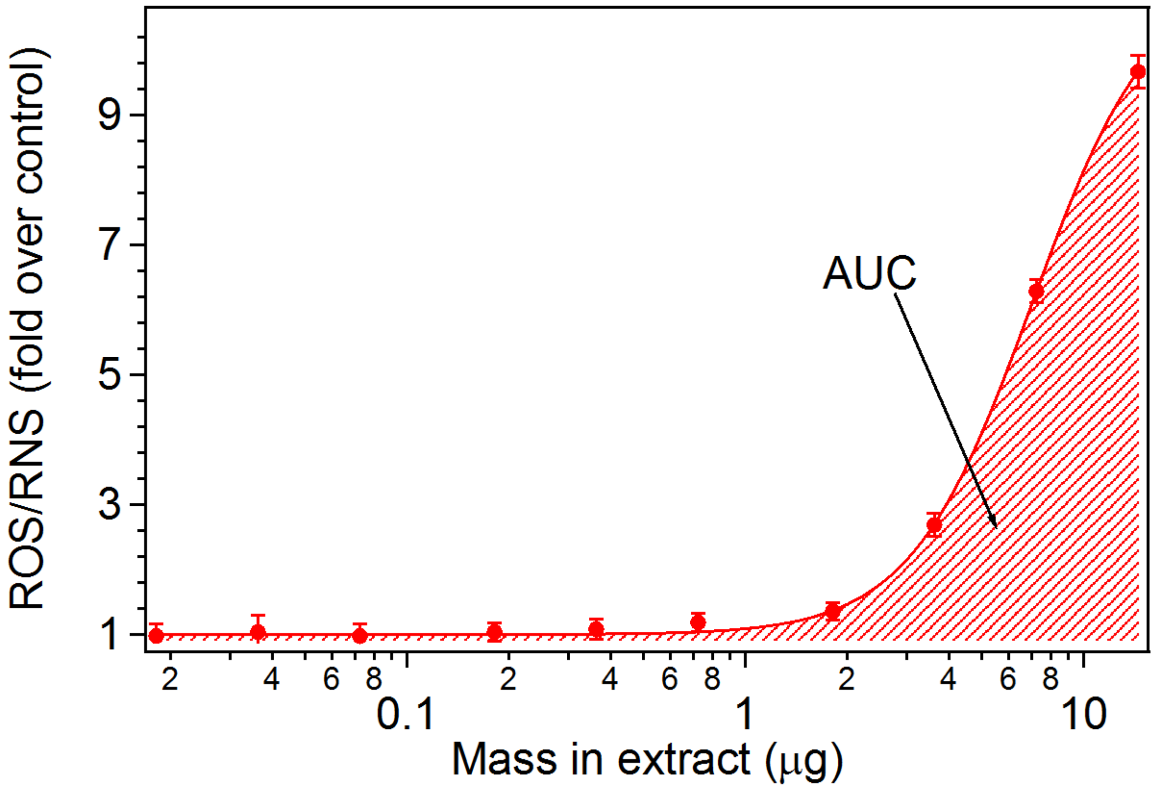

Figure S9. Representative dose-response curve of ROS/RNS produced as a result of filter exposure (Expt. 7). ROS/RNS is expressed as a fold increase over control (probe-treated cells incubated with stimulant-free media); dose is expressed as mass in the filter extract (µg). Data shown are means ± standard error of experiments performed in triplicate. The dose-response curve was fitted using the Hill equation and the area under the curve (AUC) is shown.

Table S1. Elemental ratios (O:C, H:C, N:C) as determined by AMS.

Experiment	Hydrocarbon	Seed	$[HC]_0$ (ppb)	O:C	H:C	N:C	$\overline{OS}_c$
1	naphthalene	AS[a]	32	0.78	0.99	0.015	0.58
2	naphthalene	FS[b]	32	1.59	0.77	0.012	2.4
3	naphthalene	AS[a]	92	0.56	0.98	0.012	0.13
4	naphthalene	FS[b]	84	0.78	0.98	0.014	0.58
5	naphthalene	AS[a]	186	0.48	0.98	0.010	-0.015
6	naphthalene	FS[b]	182	0.70	0.94	0.014	0.46
7	naphthalene	AS[a]	342	0.40	0.98	0.0091	-0.18
8	naphthalene	FS[b]	331	0.47	0.97	0.0093	-0.020

[a] Ammonium sulfate seed (15 mM $(NH_4)_2SO_4$); [b] Iron sulfate seed (15 mM $FeSO_4$)

References

1 Fang, T. *et al.* A semi-automated system for quantifying the oxidative potential of ambient particles in aqueous extracts using the dithiothreitol (DTT) assay: results from the Southeastern Center for Air Pollution and Epidemiology (SCAPE). *Atmos. Meas. Tech.* **8**, 471-482, doi:10.5194/amt-8-471-2015 (2015).

2 Tuet, W. Y. *et al.* Chemical oxidative potential of secondary organic aerosol (SOA) generated from the photooxidation of biogenic and anthropogenic volatile organic compounds. *Atmos. Chem. Phys.* **17**, 839-853, doi:10.5194/acp-17-839-2017 (2017).
